# Do We Need Meropenem Treatment Beyond 7 Days in Febrile Neutropenic Patients: A Cost-Effectiveness Analysis

**DOI:** 10.3390/antibiotics14070653

**Published:** 2025-06-27

**Authors:** Leyla Yumrukaya, Arnold Hagens, Ahmet Çağkan İnkaya, Gökhan Metan, Maarten Postma, Selen Yeğenoğlu, Murat Akova

**Affiliations:** 1 Department of Pharmacy Management, Hacettepe University Faculty of Pharmacy, Ankara 06100, Türkiye; selen@hacettepe.edu.tr; 2 Department of Health Sciences, University Medical Center Groningen, University of Groningen (RUG), 9700 AD Groningen, The Netherlands; arnoldjjhagens@gmail.com (A.H.); m.j.postma@rug.nl (M.P.); 3 Department of Infectious Diseases and Clinical Microbiology, Hacettepe University Faculty of Medicine, Ankara 06230, Türkiye; inkaya@hacettepe.edu.tr (A.Ç.İ.); gokhanmetan@hacettepe.edu.tr (G.M.); makova@hacettepe.edu.tr (M.A.); 4 Department of Economics, Econometrics and Finance, Faculty of Economics and Business, University of Groningen, 9700 AV Groningen, The Netherlands; 5 Center of Excellence in Higher Education for Pharmaceutical Care Innovation, Universitas Padjadjaran, Bandung 40132, Indonesia; 6 Department of Pharmacology & Therapy, Universitas Airlangga, Surabaya 60115, Indonesia

**Keywords:** cost effectiveness, febrile neutropenia, hematological malignancy, meropenem

## Abstract

Background: Febrile neutropenia is a critical complication in patients with hematological malignancies; immediate initiation of empirical treatment with a broad-spectrum agent is the standard of care. In this study, we aimed to evaluate the cost-effectiveness of long- and short-term antibiotic treatments. Methods: We conducted a retrospective cohort study. We collected data on admissions between 1 January 2018 and 31 December 2022. Adult patients treated with meropenem were included and treatment duration was categorized. Short-term treatment (STT) was defined as a period of 7 days or less, and long-term treatment (LTT) was defined as longer than 7 days. To comparatively estimate costs in both groups, it was hypothesized where STT patients were assumed to receive LTT. Three scenarios were modeled to calculate potential cost reductions. Results: In total, 151 high-risk patients were eligible, with 93 and 58 in the STT and LTT groups, respectively. Both groups exhibited similar clinical characteristics and statistically no significant differences in outcomes. The average costs for the STT and LTT groups were statistically significantly different at USD 9294.01 and USD 13,515.27, respectively. From the regression analysis, cost reductions per patient of USD 164, 527, and 690 were estimated for the three intervention scenarios. Conclusions: Notably, even though the clinical outcomes of STT were not statistically different from those of LTT, the cost of the STT group were statistically significantly lower than that of the LTT group. The early discontinuation of empirical meropenem treatment may offer financial advantages to healthcare systems.

## 1. Introduction

Febrile neutropenia (FN) is a severe condition in patients with hematological malignancies. More than eighty percent of patients undergoing intensive chemotherapy may develop FN [1]. Furthermore, patients with FN are prone to increased morbidity and in-hospital morbidity, leading to dose limitations in chemotherapy [2,3]. FN is an oncological emergency, and parenteral empirical antibiotic therapy is the standard of care in high-risk patients [3,4,5,6,7]. The risk classifications are based on the patient’s status. Regarding the Tenth European Conference on Infections in Leukemia (ECIL-10), patients are defined as “high-risk” if they have persistent neutropenia for more than 10 days, allogeneic hematopoietic stem cell transplantation, acute leukemia in induction, and relapse of refractory phase [8].

Broad-spectrum antimicrobial agents with anti-pseudomonal activity are the standard of care in the treatment of high-risk patients with FN [3,4,5,6,7,9,10,11]. Cefepime, piperacillin-tazobactam, and carbapenems (especially meropenem) are frequently recommended as antibacterial agents in the treatment of FN. Upfront carbapenem use is usually reserved for those admitted with sepsis or septic shock, with known colonization or previous infection with extended-spectrum beta-lactamase (ESBL)-producing gram-negative bacilli [8]. However, the duration of empirical therapy in those patients with fever of unknown origin who defervesce after empirical antibacterial therapy is debatable. While the Infectious Diseases Society of America guidelines indicate a risk assessment-based approach for treatment duration, the ECIL-10 guidelines suggest discontinuation of antibiotic treatment after 72 h if patients are afebrile for the last 48 h, irrespective of recovery from neutropenia [7,8].

Apart from the clinical outcomes, prolonged treatments are also related to financial outcomes, particularly in conditions such as FN, where extended hospitalization and intensive antimicrobial therapy can result in increased costs [12,13,14]. Although the burden of cancers and the cost of various agents have been examined in the existing literature, there are no studies on the real-world costs and effectiveness of different treatment durations of empirical meropenem treatment in FN. Thus, in this study, we aimed to compare the costs of meropenem regarding the duration of treatment in FN patients with hematological malignancy or stem cell transplantation, in the context of potential similar clinical outcomes as suggested by clinical guidelines.

## 2. Results

### 2.1. Patient Characteristics

**Figure 1:**
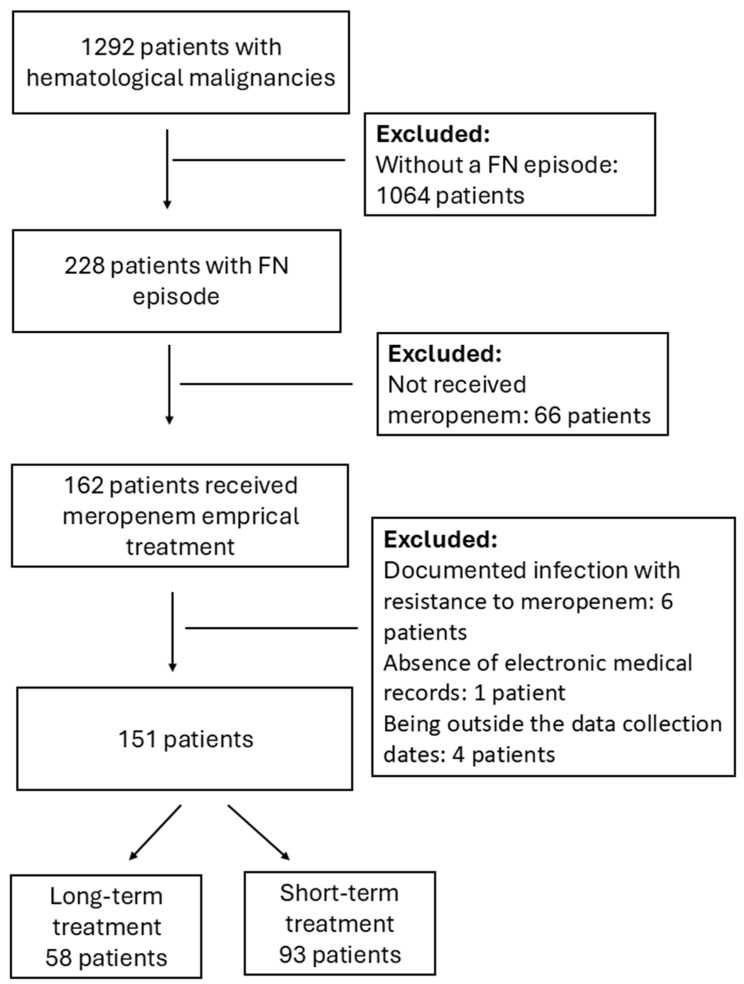
Study flowchart based on inclusion/exclusion criteria.

A total of 1292 patients with hematological malignancies were screened, 228 of whom had FN episodes. A total of 162 patients received meropenem as empirical treatment. Of these, 11 were excluded based on the exclusion criteria. Finally, 151 patients were included: 93 in the short-term treatment (STT) group and 58 in the long-term treatment (LTT) group. (Figure 1).

All patients were Caucasian inpatients admitted to hospital wards and deemed to be “high-risk”. The most common underlying disease was acute myeloid leukemia, followed by non-Hodgkin’s lymphoma and acute lymphoid leukemia [39.7% (n = 60), 17.2% (n = 26), 13.20 (n = 20)]. Most patients were either newly diagnosed or had recurrent disease [21.9% (n = 33), 21.9% (n = 33)]. A total of 55 underwent stem cell transplantation of which 34 were autologous transplantations. [22.5% (n = 34), 13.9% (n = 21)]. A total of 106 patients had one episode of neutropenic fever, and 70 were in the short-term arm. Both groups exhibited comparable demographic and diagnostic characteristics (Table 1).

### 2.2. Outcome Analysis

In both arms, most patients experienced only one neutropenic fever. The primary endpoints were comparable between the arms (Appendix A). The average hospitalization period was 42.04 days, and the mean neutropenia duration was 13.47 days. During the first episode of FN, fever was observed for an average of 2.59 days. The average number of antibiotic-free days during hospitalization was 23.42 days. The hospitalization duration was statistically significantly different between the two arms (*p* < 0.05). Moreover, statistically significant differences were found between the number of febrile days, total number of febrile days, and fever-free days in the first episode between the two groups (*p* < 0.05). In addition to the clinical outcomes, the average cost was USD 10,915.42 for all patients. The average cost for the short-term arm was USD 9294.01; for the long-term arm, it was USD 13,515.27 because group sizes differ, which is a lower cost per patient. The difference in costs between the two arms was found to be statistically significant. (*p* < 0.05). The r values showed that the effect strength is medium for febrile days in first episode and febrile days during hospitalization, and small for hospitalization, afebrile days during hospitalization and costs (Table 2).

### 2.3. Cost Analysis Results

The additional drug costs and hospital stay costs were estimated based on the difference in treatment duration and hospitalization duration between the two groups (7.4 and 8.6, respectively). As health outcomes are assumed not to change, cost savings are expected without negatively affecting the quality of life. The regression analysis yielded an estimated cost of USD 99.88 per additional hospitalization day and USD 35.99 for an additional treatment day. However, the adjusted R^2^ yielded a value of 0.083 and 0.06, respectively, showing that a part of the variance in the costs still remains to be explained. In addition, costs were estimated using the management-reported cost per hospitalization day at USD 13.75 per day and the mean drug cost per treatment at USD 30.12 per day. For both approaches, the cost estimation results are presented in Table 3. For the first approach, cost reductions per patient are USD 164, 527, and 690 for the three intervention scenarios. In the second approach, the cost reductions are USD 137, 72, and 210 per patient, respectively.

As the linear regression might have overestimated the extra costs because the daily cost for STT patients was likely reduced due to the improving state, it is reasonable to believe that real cost advantages are between the two calculation methods, and therefore, savings may lie between USD 137 and USD 690 per day per patient.

## 3. Discussion

In this cost-effectiveness study, we compared the long- and short-term meropenem treatment for FN patients with hematological malignancies. We observed that long- and short-term treatments resulted in similar clinical outcomes with decreased cost in the short-term treatment arm. Notably, without additional drug and hospitalization costs, costs decreased by USD 210–690 per patient. Considering that a one-night hospital stay cost is USD 3.20 in Türkiye, the cost reduction can be interpreted as 65–215 days of savings per patient.

Empirical antibiotic treatment is the mainstay of FN management; however, the optimal duration remains controversial. Notably, the ECIL has emphasized the challenges of increasing the risk of antimicrobial resistance and narrowing or minimizing the empirical use of carbapenems [3]. However, increasing rates of bloodstream infections caused by third-generation cephalosporin-resistant gram-negative bacilli in FN patients make carbapenems the first-line treatment [15,16]. Besides, prolonged antibiotic treatment leads to antimicrobial resistance, which increases the burden of FN [17,18]. Thus, the optimization of antibiotic treatment duration is an urgent issue in the growing era of antimicrobial resistance [19]. The clinical outcomes and safety of short-course antimicrobial treatments have been addressed in previous studies in different patient populations considering de-escalation and discontinuation [17,20,21,22,23,24,25,26]. In a randomized open-label study, short-term treatment periods were determined to be non-inferior to long-term ones [20]. In a systematic review of eight randomized clinical trials, there were no statistically significant differences in all-cause mortality rates between short-term treated patients [18]. Although these studies have different methodological aspects, they have highlighted the benefits and safety of short-course antimicrobial treatment. In a systematic review, Ishikawa et al. indicated that the efficacy of short- and long-term treatment periods in FN did not differ significantly [27]. Similarly, Niessen et al. found no difference in mortality associated with the duration of antibiotic therapy [23].

Apart from the clinical aspects, malignancies are a major financial burden in the healthcare system. Hematological malignancies are a major contributor to the global burden, whereas leukemia has the highest burden [28]. Although specific studies on expenditures in hematological malignancies are limited, more than half of the expenditures related to cancer are reported as hospitalization costs, and approximately one-third of them are drug expenditures. In addition to the duration of treatment, a significant portion of the drugs used in cancer treatment are new treatments and this situation causes higher drug costs [29]. Moreover, Burns et al. showed that in five European countries with the highest populations, expenditures from hematological malignancies accounted for a large proportion of cancer-related health expenditures [30].

Notably, hospitalization days are the main drivers of oncological hematological diseases [31,32]. A decrease in hospital stay is directly associated with a decrease in direct medical costs [33]. Furthermore, FN also affects health expenditures in hematological malignancies [12]. As a frequent complication of hematological malignancies, FN requires additional treatment and increases the length of hospitalization [13,14]. In our study, the number of hospitalization days was lower in the short-term arm than in the long-term arm (<0.05). Fewer hospitalization days in the STT group is attributable to the clinical situation of the patients. Following induction and more commonly consolidation chemotherapy, particularly in patients who are in remission, the primary cause for hospitalization is antibiotic treatment for FN. In this case, the shorter hospitalization days are directly associated with shorter antibiotic treatment.

Next to hospitalization, as requiring additional treatments, FN is also related to increases in drug expenses. Considering carbapenem use, Tori et al. showed that meropenem has the highest cost when comparing different agents [33]. Besides, prolonged antibiotic treatment is associated with longer hospitalization, and as for adverse effects, secondary infections, *Clostridioides difficile* colitis, and toxicities [27]. Although we did not observe excess adverse events in our cohort, we still defined cost savings in the short antibiotic duration group. In addition, more hospitalization stays also increase the burden on nursing services. As Türkiye has one of the lowest numbers of nurses, the decrease in hospitalizations will also contribute to the effective use of the nurse health workforce [34].

In this study, although we have worked on the subject of the cost-effectiveness of the duration of empirical treatment in FN patients with hematological malignancies, it should not be ignored that our research has some limitations. First, we conducted a single-center study; therefore, the sample size is inherently limited, which may impact the detection of statistical differences. Secondly, all data were extracted from an electronic medical database retrospectively. Even though the records were detailed, we could have possibly missed some factors that could affect outcomes due to time constraints. Moreover, we only included direct medical costs from the payer’s perspective because of the retrospective approach. Thus, financial burdens such as productivity losses and caregiver burden could not be interpreted. Thirdly, because we obtained data from electronic medical records, information bias could not be negligible. Moreover, we included only meropenem treatments; therefore, patients taking other agents were excluded. Although evaluating a single agent within a single center provides internal consistency, it limits generalizability. Cost-effectiveness analyses for other empirical agents should take into account their specific use contexts. Factors such as the need for escalation, clinical setting, and the degree of interdisciplinary collaboration in FN management may significantly influence outcomes. Therefore, these variables may limit the generalizability of our findings to other empirical treatments used in different agents within different centers. Furthermore, since the hospital was designated as a COVID-19-free facility, the effects of COVID-19 could not be interpreted in this study. Lastly, the average costs per hospitalization day and treatment day were estimated using a regression model. However, the model yielded with a low R^2^ value. The large proportion of variance in the costs remains unexplained, potentiallyaffecting of the estimated the costs per treatment and hospitalization day.

## 4. Materials and Methods

### 4.1. Study Design

This was a single-center retrospective cohort study that compared the direct medical costs of short versus long meropenem treatment durations in patients with hematological malignancies who experienced their first FN episode during hospitalization. The study was conducted at the Hacettepe University Adult Hospital in Ankara, including admissions between 1 January 2018 and 31 December 2022. The date restrictions were determined to obtain patients’ accessible data. High-risk patients aged 18 years and over, treated with meropenem, were included in the study. Patients were categorized per their age as 65 years of age or older and younger than 65 years of age considering risk stratification.

Considering the local guideline, the indications in which meropenem was used as the first-line agent were as follows: previous colonization or infection with extended-spectrum beta-lactamase (ESBL) producing gram-negative bacilli or multidrug-resistant gram-negative bacilli (characterized with resistance to at least one agent in three or more antibiotic classes); presence of septic shock (sepsis with persistent hypotension requiring vasopressors to maintain a mean arterial pressure exceeding 65 mm Hg and a serum lactate level exceeding 2 mmol/L despite adequate volume resuscitation); nosocomial pneumonia, FN developed under quinolone prophylaxis; or history of receipt of broad-spectrum antibiotics within 10 days, or history of hospitalization in the intensive care unit for more than 72 h within the last 15 days [15]. Patients with septic shock at the beginning of treatment, clinically and/or microbiologically demonstrated foci of infection, infected or colonized with carbapenem-resistant bacteria, or positive blood cultures requiring continuation of systemic antibiotic treatment were excluded.

A total of 1292 patients with hematological malignancies were admitted to the hospital. In total, 228 patients had febrile neutropenia episodes and of these, 66 patients were excluded as they were not treated with meropenem. A total of 162 patients were treated with meropenem and 11 patients were excluded. In total, 6 of them had documented infection with resistance to meropenem, 1 patient had no electronic medical record, and 4 patients were outside of the data collection period. Finally, 151 patients were included in the study, 58 patients in the long-term group, 93 patients in the short-term group. The flow chart is presented in Figure 1.

### 4.2. Definition of Febrile Neutropenia

FN was defined as a single tympanic temperature of ≥38.3 °C, or a temperature of ≥38 °C for at least 1 h with absolute neutrophil count (ANC) of less than 0.5 × 10^9^/L, or an ANC expected to decrease below 0.5 × 10^9^/L in the next 48 h [7].

### 4.3. Comparison Groups

The comparison was based on the treatment duration of the patients who received meropenem during the first episode of FN. Our local study on adherence to the FN protocol showed a statistically significant decrease in the number of patients receiving seven or fewer days of treatment after protocol implementation [35]. Using a 7-day threshold, we aimed to demonstrate that shorter treatment durations can be a cost-effective, efficient, and safe approach. Notably, Ishikawa et al. reported that studies define short duration as 7 days [27]. Based on our local data and existing literature, we used 7 days as a cut-off point. Accordingly, STT was defined as a period of 7 days or less, and LTT was defined as a period longer than 7 days.

### 4.4. Outcomes

The outcomes were determined as the primary endpoints based on the study design. The outcomes to be compared in line with treatment durations were as follows: number of recurrent episodes of neutropenic fever, requirement of intensive care unit transfer, infection-related mortality, initiation of antibiotics within 48 h after meropenem discontinuation, detection of bloodstream infection at least 24 h after starting meropenem and within 14 days, escalation of meropenem treatment, 30-day mortality, *Clostridioides difficile* infection, fungemia, rate of invasive pulmonary aspergillosis development, number of intravenous antibiotics-free days.

### 4.5. Data Collection

The selection of patients and related information were obtained from the electronic medical records database of the Hacettepe University Adult Hospital. Subsequently, the records of each patient were screened, visit notes were examined in detail, and all diagnostic tests, including laboratory tests, were extracted from these records.

### 4.6. Costs

The payer perspective was set a priori; therefore, the direct medical costs were included. Costs were calculated using a top-down approach and obtained from the billing records. Next, they were adjusted for inflation and converted in 2024 USD values using the appropriate exchange rates. The adjusted value was calculated by multiplying the original value with the consumer price index ratio which was derived by dividing the 2024 consumer price indexes in each year.

### 4.7. Cost Analysis

To estimate the cost savings of including an STT strategy, we considered the baseline with all patients being eligible for long-term treatment. Ergo, we constructed a hypothetical baseline in which patients with STT were assumed to undergo LTT treatment. The health outcomes of STT were assumed to be the same as if they had undergone LTT. However, costs were assumed to increase by shifting from STT to LTT for the two main drivers: drug treatment and hospital stay. The cost of drugs would increase when patients with STT are shifted to LTT, as antibiotics will not be stopped. Therefore, the additional drug costs would be equal to the number of extra treatment days multiplied by the cost of drugs per treatment day. This would also be the case for the cost of hospitalization and leading to an extra hospitalization cost equal to the additional stay multiplied by the cost of an additional hospitalization day. We evaluated the cost savings with two approaches. In the first approach, we used regression coefficients, and in the second approach, we used mean drug costs and reported hospitalization costs as they reflect the real resource use and costs better than medians.

Other costs were not included, as the STT patients were assumed not to require any additional costs, such as those related to intensive care unit. In the first approach, hospitalization cost was estimated based on the mean difference in hospitalization days, which was 8.6 days less for STT than LTT, multiplied by the unit cost of an additional hospitalization day. The cost of an additional hospitalization day was estimated using a straightforward linear regression model including variables obtained from patient records possibly affecting the costs (Appendix B). Variables with low variation were left out. Multicollinearity was not observed showing that the independent variables were not correlated. No additional analysis on confounding variables was done. This approach was chosen over the mean calculation, which would have led to an overestimation of the additional daily hospitalization costs by including expenses no longer applicable to nearly recovered STT patients. The dependent variable was the total hospitalization cost minus the cost of drugs. The additional drug costs were similarly estimated based on the mean difference of treatment days at 7.4 days less, multiplied by the unit cost of an additional treatment day. The cost of an additional treatment day was also estimated through linear regression (Appendix B).

Since using regression analysis might overestimate the additional cost for hospitalization and treatment for STT patients, we also used the second approach. In the second approach, the additional hospitalization and drug costs were also estimated using the management-reported cost per additional hospitalization day (including bed cost and two physician visits) and the mean drug cost per additional treatment day. As a result, we were able to gain more insight into the cost-savings range.

Hospitalization and drug costs were also taken into account as they are the main costs drivers. Thus, in addition to the hypothetical baseline, we conduct three different scenarios considering the main cost drivers. The first scenario does not include the extra drug costs for the STT group. The second excludes only the extra costs for the hospitalization of the STT group, and the third scenario excludes the additional drug costs and hospitalization costs. Through these three intervention scenarios, we can gain insights into the possible cost savings of short-term treatments.

### 4.8. Statistical Analysis

The complete anonymized data were analyzed using the IBM Statistical Package for Social Science (SPSS) version 23.0 (IBM Corp., Armonk, NY, USA). After descriptive analysis, the t-test was used in cases where the parametric test assumptions were met, and the Mann–Whitney U test was used in cases where they were not. Chi-square tests were done to analyze categorical data related to the sample. The r value was calculated to present the effect size by dividing the Z value to the square root of the sample size. R version 4.3.3 was used for the linear regressions conducted for the cost estimates.

## 5. Conclusions

In conclusion, the clinical outcomes of short-term treatment were not significantly different from those of long-term treatment alone. Although the existing literature focused on the clinical aspects, in our study we found that the cost of the short treatment arm was lower than that of the long treatment arm with similar clinical outcomes. Even though this is the first study that presents different treatment durations and their costs, the results will be beneficial for institutional practices. Moreover, the early discontinuation of empirical antibiotic treatment with meropenem may offer advantages in terms of healthcare costs in addition to antimicrobial stewardship practices. Yet, changes in nationwide policies will require more studies.

## Figures and Tables

**Table 1:** Comparison of patient characteristics.

Characteristics	n (%)	STT (n = 93)	LTT (n = 58)	*p* Value
Age				
<65	136 (90.06)	84	52	0.894
≥65	15 (9.94)	9	6	
Sex				
Male	80 (52.98)	48	32	0.670
Female	71 (47.02)	45	26	
Primary diagnosis				
Acute lymphoid leukemia	25 (16.60)	17	8	0.261
Acute myeloid leukemia	60 (39.70)	35	25	
Hodgkin lymphoma	5 (3.30)	5	0	
Non-Hodgkin lymphoma	26 (17.20)	18	8	
Multiple myeloma	20 (13.20)	12	8	
Chronic leukemia	2 (1.30)	1	1	
Other	13 (8.60)	5	8	
Diagnosis classification				
New	33 (21.90)	18	15	0.288
Recurrent	33 (21.90)	25	8	
Remission	71 (47.00)	40	31	
Residual disease	8 (5.30)	6	2	
Refractory	6 (4.00)	6	2	
Co-morbidities				
None	106 (70.20)	69	37	0.317
Lung diseases	5 (2.60)	3	1	
Heart diseases	20 (6.60)	5	5	
Renal diseases	2 (1.30)	1	1	
Liver diseases	6 (4.00)	4	2	
Diabetes mellitus	17 (11.30)	10	7	
Immunodeficiency other than the primary malignancy	6 (4.00)	1	5	
Bone marrow transplantation				
None	66 (63.60)	61	35	0.482
Autologous	34 (22.50)	18	16	
Allogeneic	21 (13.90)	14	7	

**Table 2:** Comparison of outcomes and effect strengths.

Outcomes	STT (≤7 Days) (n = 93) (Min. to Max.)	LTT (>7 Days) (n = 58) (Min. to Max.)	*p* Value (Confidence Intervals)	r Value
Neutropenia duration (days)	13.13 (1 to 100)	14.02 (1 to 43)	0.080 (−5.00, ~0)	-
Hospitalization (days)	38.75 (4 to 117)	47.31 (13 to 140)	**0.005** [−15.0, −3.0]	0.23
Febrile days in first episode	1.87 (1 to 15)	3.74 (1 to 13)	**0.000** [−2.0, −1.0]	0.37
Febrile days during hospitalization	3.37 (1 to 44)	6.57 (1 to 59)	**0.000** [−3.0, −1.0]	0.40
Afebrile days during hospitalization	35.29 (2 to 111)	40.84 (11 to 111)	**0.036** [−12.0, ~0]	0.17
Number of intravenous antibiotics-free days	24.42 (0 to 111)	21.83 (1 to 75)	0.789 [−3.0, 4.0]	-
Days between two episodes	4.89 (0 to 60)	2.72 (0 to 57)	0.467 [~0, ~0]	-
Cost (USD)	9294.01 (108,894.68 to 99,765.51)	13,515.27 (101,463.90 to 92,629.18)	**0.013** [−5.4, −631]	0.20

**Table 3:** Cost analysis for the scenarios for two estimation methods (in USD).

Scenario	Approach 1: Using Regression Coefficient for Additional Costs			Approach 2: Using Mean Drug Costs and Reported Hospitalization Cost		
	Per Patient	Incremental Costs Compared with Baseline	Total Costs (N = 151)	Per Patient	Incremental Costs Compared with Baseline	Total Costs (N = 151)
Baseline is not an intervention scenario	11,608		1,752,738	11,127		1,680,138
Without additional drug costs	11,444	−164	1,728,003	10,990	−137	1,659,437
Without additional hospitalization cost	11,081	−527	1,673,226	11,054	−72	1,669,192
Without additional drug and hospitalization cost	10,917	−690	1,648,491	10,917	−210	1,648,491

## Data Availability

The data presented in this study are available on request from the corresponding author.

## Abbreviations

The following abbreviations are used in this manuscript:

USD	American Dollar
FN	Febrile neutropenia
STT	Short-term treatment
LTT	Long-term treatment
ESBL	Extended-spectrum beta-lactamase
ECIL-10	Tenth European Conference on Infections in Leukemia

## Appendix A

The outcomes comparison for two treatment groups is presented in Table A1.

**Table A1:** The outcomes comparison for two treatment groups.

Outcomes	n (%)	STT (≤7 Days)	LTT (>7 Days)	*p* Value
Number of neutropenic fever episodes				
1	106 (70.2)	70	36	0.206
2	30 (19.9)	16	14	
More than 2	15 (9.9)	7	8	
Requirement of transfer to intensive care unit				
None	133 (88.1)	85	48	0.092
Yes	18 (11.9)	8	10	
Infection-related mortality				
None	143 (94.7)	90	53	0.144
Yes	8 (5.3)	3	5	
Initiation antibiotics within 48 h after antibiotic discontinuation				
None	116 (76.8)	69	47	0.222
Yes	35 (23.2)	24	11	
Proliferation in blood culture 24 h after carbapenem initiation and within 14 days				
None	130 (86.1)	80	50	0.588
Yes	21 (13.9)	13	8	
Gram-negative bacteria growth in blood culture in 48 h				
None	116 (76.8)	72	44	0.488
Yes	21 (13.9)	21	14	
Bacterial growth in other cultures				
None	118 (78.1)	72	46	0.475
Yes	33 (13.9)	21	12	
Invasive pulmonary aspergillosis development				
None	140 (92.7)	88	52	0.336
Yes	11 (7.3)	5	6	
Fungemia development				
None	149 (98)	93	56	0.146
Yes	2 (1.3)	0	2	
Clostridioides difficile infection development				
None	148 (98)	92	56	0.559
Yes	3 (1.3)	1	2	
30-days mortality				
None	144 (95.4)	91	53	0.107
Yes	7 (4.6)	2	5	
Escalation				
None	146 (96.69)	89	57	0.656
Yes	5 (3.31)	4	1	

## Appendix B

Regression results are presented in Table A2, Table A3 and Table A4.

**Table A2:** Regression results.

Regression Results			
	Dependent Variable:		
	USDollarsWOcarbo		
	ALL	STT	LTT
Sex 1: Male, 2: Female	880.399	−295.156	1286.713
	(1641.383)	(1880.539)	(3127.992)
Age	−40.411	−16.168	−16.781
	(54.347)	(59.703)	(109.321)
Comorbidity	803.196 **	832.417	596.193
	(389.569)	(519.582)	(637.515)
Neutropenic days	−55.408	−13.064	−376.407 *
	(69.073)	(72.207)	(192.459)
Hospitalization days	133.222 ***	99.879 **	257.415 ***
	(36.901)	(38.385)	(80.227)
Treatment duration in days	−136.797	−21.835	−216.348
	(258.229)	(708.630)	(353.355)
Febrile days	−24.204	387.787 *	−237.213
	(144.755)	(223.028)	(207.494)
Episode number	−521.304	−2667.120	828.241
	(1577.805)	(2004.542)	(2676.825)
ICU need	−2062.147	−6231.693 *	−685.228
	(2743.710)	(3477.747)	(4589.845)
Short Long Term 1: short, 2: long	3859.023		
	(2449.955)		
Constant	2309.156	8705.241	7410.392
	(4736.650)	(6622.957)	(8444.803)
Observations	151	93	58
R^2^	0.165	0.173	0.247
Adjusted R^2^	0.105	0.083	0.106
Residual Std. Error	9618.904 (df = 140)	8383.558 (df = 83)	11,105.330 (df = 48)
F Statistic	2.757 *** (df = 10; 140)	1.925 * (df = 9; 83)	1.750 (df = 9; 48)
Note:	* *p* < 0.1; ** *p* < 0.05; *** *p* < 0.01		

**Table A3:** Regression results for drug costs.

Regression Results Meropenem Costs			
	Dependent Variable:		
	Mero_Cost_Dollars		
	ALL	STT	LTT
Sex 1: Male, 2: Female	−15.778	−33.791	−2.726
	(31.488)	(49.019)	(28.264)
Age	0.051	0.978	−2.198 **
	(1.043)	(1.556)	(0.988)
Comorbidity	3.369	−7.729	9.464
	(7.473)	(13.544)	(5.760)
Neutropenic days	−0.848	−1.313	0.244
	(1.325)	(1.882)	(1.739)
Hospitalization days	0.761	1.566	−1.365 *
	(0.708)	(1.001)	(0.725)
Treatment duration in days	26.862 ***	35.990 *	33.105 ***
	(4.954)	(18.471)	(3.193)
Febrile ddays	−3.570	−3.084	−2.823
	(2.777)	(5.814)	(1.875)
Episode number	14.808	13.301	36.557
	(30.268)	(52.251)	(24.187)
ICU need	133.561 **	206.149 **	59.316
	(52.634)	(90.652)	(41.473)
Short Long Term 1: short, 2: long	−56.007		
	(46.999)		
Constant	53.266	−86.844	−1.386
	(90.866)	(172.636)	(76.305)
Observations	151	93	58
R^2^	0.363	0.152	0.755
Adjusted R^2^	0.318	0.060	0.709
Residual Std. Error	184.526 (df = 140)	218.528 (df = 83)	100.345 (df = 48)
F Statistic	7.990 *** (df = 10; 140)	1.650 (df = 9; 83)	16.400 *** (df = 9; 48)
Note:	* *p* < 0.1; ** *p* < 0.05; *** *p* < 0.01		

**Table A4:** Variance inflation factors of both regression analyses.

Independent Variable	VIF
Sex 1: Male, 2: Female	1.10
Age	1.07
Comorbidity	1.08
Neutropenic days	1.51
Hospitalization days	1.74
Treatment duration in days	2.66
Febrile days	1.84
Episode number	1.78
ICU need	1.29
Short Long Term 1: short, 2: long	2.32
